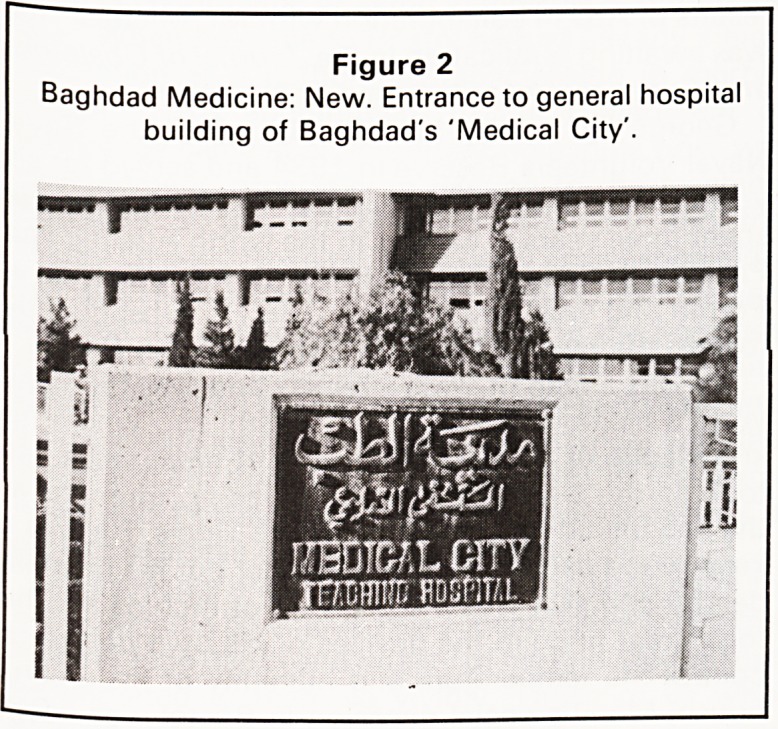# From Our Foreign Correspondent

**Published:** 1985-07

**Authors:** 


### From our Foreign Correspondent

### Baghdad: Old, New & Us

*To enter into old age through the gate of infirmity is most disheartening.*
*Horace Walpole (1765)*[1]

Prophets of doom have said for a long time that Britain is past its genteel middle age. With the loss of Empire and the decline in manufacture we are a fading beauty. Is this how others see us?

Last winter an invitation to the English Royal College of Surgeons was extended by the Iraqi Ministry of Health to examine local candidates for the Primary FRCS. By chance, a junior examiner—your temporarily Foreign Correspondent—was asked to join the party. Happily it transpired that his wife, who is in catering was invited too and was able to come as well.

Not being used to such treats we accepted with alacrity. Older and wiser colleagues were given to mentioning gently that there was an Armed Conflict in that Part of the World. Our innocent response was merely to buy antimalarials from Boots the Chemists, and to think vaguely of updating jabs against infections. Who was right? Both parties, one can say.

What was gained by going? After all, anyone could have foretold that today's rigours of unexpected airport searching (lest you are a hi-jacking terrorist's Moll) and military street arrest (just in case your 35 mm camera slung under a jacket was really a waist-level gun holster) were fair risks. They in fact became reality but were not, in perspective, experiences that marred the trip. We had wanted to go in any case; troubles or no.

Next let me say that it was our dim impression from schooldays that the valley of the Tigris and Euphrates gave rise to City Life. Second, we realised that both medicine and cooking have a long history in Mesopotamia, and that Baghdad was an important centre in their development. University-type teaching also developed here: one ancient gate to learning is shown in Figure 1. Finally we appreciated (partly through last-minute perusal of a neglected set of Gertrude Bell's Letters to her family[2]) that there has been a long-standing Anglo-Iraqi connection. The visit confirmed all these intimations. The city buildings, old and new, even aeroplane security, our personal and cultural experiences in Baghdad showed that the various warnings and encouragements were correct. However it must be said that we felt an awed respect for what had been achieved in the distant and in the recent past in Mesopotamia. The climate is not easy: summer temperatures in the region of 120 °F are not easily borne[2] (the local hospital admissions for heat-stroke today support this fact).

**Figure 1**
Baghdad Medicine: Old. The gate and severe rectangular walls protect the Mustansiriya College, which dates from the 12th c. A.D.

What else is there to say? As usual, the most predictable impressions from foreign travel are those that spring from problems common to the visitor and his hosts. For a start, the Examination Candidates ranged from the diffident (they left the scene remarkably early) to the impressively knowledgeable. Caterers in hospital and commercial posts, surgeons and pathologists, all seemed to be of reassuringly familiar moulds. There was no difficulty in understanding shared frustrations and pleasures with any of them. Clearly, however, local variations were evident. Seasonal excesses (or deficiencies) in culinary commodities, and geographical differences in pathology (I personally had not seen so much hydatid disease in 20 years of English laboratory work as in a twenty-minute clinical Ward Round) were also to be expected. All these aspects were soon revealed in the modern 'Medical City' of Baghdad (Figure 2), where we were cordially received by our opposite numbers. Established and modern approaches obviously both have their place in catering and pathology in England and Iraq. The problems are very similar.

**Figure 2**
Baghdad Medicine: New. Entrance to general hospital building of Baghdad's 'Medical City'.

The unexpected lies in this. What training is the most appropriate? And for whom? Whilst working in this country we implicitly feel that a British attitude to training (medical or anything else) is best. For us, yes may be. There are in support of such a view, plenty of FRCS, MRCOG, and MRCP (UK) Display Boards proudly hanging in the streets of Baghdad. It should be said, however, that Cooks and Institutional Managers are more restrained in this respect. And yet, medical American Board certificants, and caterers trained in Asia and on continental Europe are fast becoming much more numerous in the Middle East than are those of English origin. Might it possibly be that British training is merely one of the many choices for the Rest of the World?

When here in the U.K. this might seem a daft question. But is it? Who else thinks this a solecism? Not many, it seems.

On return one is horrified to see once more the further and progressive financial erosion of our own training schemes. The current cut-backs in the British Council, University under- and post-graduate courses, and even of professional post-graduate training activities seem counter–productive after Setting Foot Elsewhere. If we cannot provide training, you may rest assured that there are plenty of others in the United States, Europe, Russia, and now in the Arab countries themselves who will. To be frank, one fears that British attitudes in these fields may sound an historical turn of the circle. In the not-so-distant future we may have to return to the Middle East: for our own training. Remember the early Middle Ages? It will be unfortunate should Islam decide to charge Full Rates. Such charges were not the basis of the early Muslim universities,[3] which in any case antedate the current foundations in north and western Europe. Even more relaxed photocopying laws will not allow for reproduction of the enormous libraries that once were established in Baghdad and Alexandria. We cannot rely upon Mongolian hordes to sack the modern libraries this time round in the Middle East.

Surely we do not want a further decline in British medical influence. Infirmity is not only physical: finances and parochialism can also lead to Walpole's gate. Such an enforced old age would certainly be disheartening.

JACK DAVIES
Department of Pathology
University of Bristol

### REFERENCES

1. WALPOLE, H. (1765). Letter to Mr. G. Montagu, from Strawberry Hill, Twickenham. *Letters of H. Walpole*, Ed. P. Cunningham, *John Grant, Edinburgh*, 1906, vol. 4, pp. 385–387.
2. BELL, G. (1927). *The Letters of Gertrude Bell*, Ed. Lady F. Bell. *E. Benn, London*, 2 volumes.
3. GUILLAUME, A. (1931). Philosophy and theology. In *The Legacy of Islam*, Eds. T. Arnold and A. Guillaume. *Oxford University Press, London*, p. 241.

## Figures and Tables

**Figure 1 f1:**
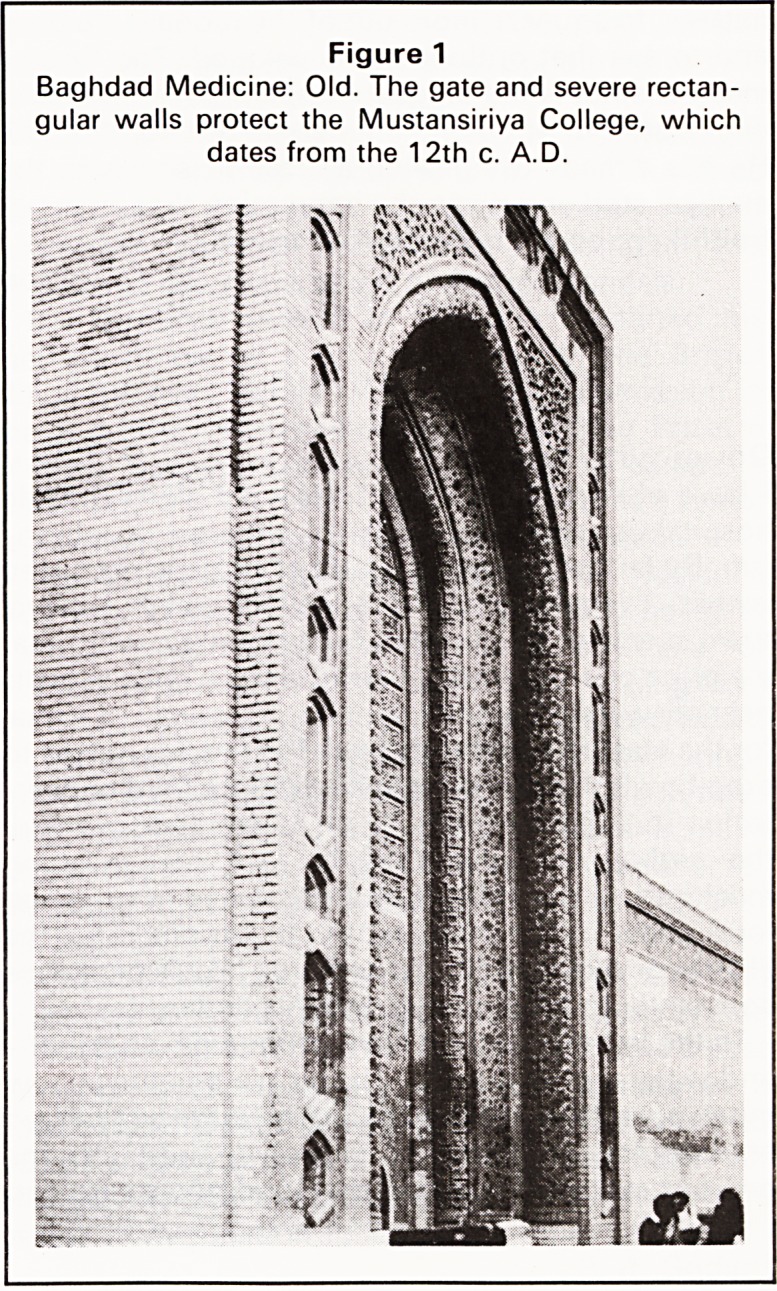


**Figure 2 f2:**